# A retrospective review of small intestinal intussusception in 126 cattle in Switzerland

**DOI:** 10.1002/vro2.58

**Published:** 2023-03-28

**Authors:** Ueli Braun, Christian Gerspach, Claudia Volz, Muriel Boesiger, Monika Hilbe, Karl Nuss

**Affiliations:** ^1^ Department of Farm Animals Vetsuisse Faculty University of Zurich Zurich Switzerland; ^2^ Institute of Veterinary Pathology Vetsuisse Faculty University of Zurich Zurich Switzerland

## Abstract

**Background:**

Intussusception is a form of ileus of the intestines in which an oral intestinal segment slides into the adjacent aboral intestinal segment, causing obstruction of the bowel.

**Methods:**

We analysed the medical records of 126 cattle with intussusception of the small intestine.

**Results:**

Demeanour and appetite were abnormal in 123 cattle. Non‐specific signs of pain occurred in 26.2%, signs of visceral pain in 46.8% and signs of parietal pain in 56.4%. Intestinal motility was decreased or absent in 93.7% of the cattle. The most common findings of transrectal palpation were rumen dilation (37.3%) and dilated small intestines (24.6%). In 96% of the cattle, the rectum was empty or contained little faeces. The principal laboratory findings were hypokalaemia (89.6%), hypocalcaemia (76.5%), base excess (72.9%), hypochloraemia (71.8%), azotaemia (62.1%) and haemoconcentration (61.1%). The main ultrasonographic findings were reduced or absent intestinal motility (98.2%) and dilated small intestines (96.0%). A diagnosis of ileus was made in 87.8% and a diagnosis of ileus attributable to intussusception was made in another 9.8%. Right‐flank laparotomy was carried out in 114 cattle. Fifty‐six (44.4%) cows were discharged.

**Conclusions:**

Clinical findings of intussusception in cattle are often non‐specific. Ultrasonography may be required to diagnose ileus.

## INTRODUCTION

Intussusception is a form of mechanical ileus of the small or large intestine that occurs when an oral intestinal segment slides into an aboral segment in a telescope‐like fashion, causing obstruction of the bowel.^1^ Four types of intussusception have been described: jejunojejunal/jejunoileal, ileocecocolic, cecocolic and colonic.^2^ The most common type is jejunoileal intussusception.^1^ The clinical and laboratory findings of cattle with intussusception have been described in textbooks,^1^, ^2^, ^3^, ^4^ continuing education materials^5^ and publications that involved 15,^6^ 20^8^ 38 and 57 cases,^2^ 21 respectively. Numerous case reports of cattle with intussusception have been published,^7^, ^8^, ^9^, ^10^, ^11^, ^12^, ^13^, ^14^, ^15^, ^16^, ^17^, ^18^, ^19^, ^20^ while older studies (1917–1964) have been reviewed.^21^ Several studies involved percutaneous ultrasonographic imaging of intussusception via the right flank in cows,^17^, ^22^, ^23^, ^24^, ^25^, ^26^ and one study used transrectal imaging.^27^

Early clinical signs of intussusception include abdominal pain, progressive inappetence, listlessness, reduced or no faecal output, dilated abdomen and dehydration.^2^, ^5^, ^6^, ^28^ Residual faeces in the rectum may be dark and contain blood or mucus.^1^ The heart rate increases with increasing abdominal pain, intestinal necrosis and dehydration.^3^ Intussusception typically has a three‐phase course.^1^ Phase 1, also referred to as the colic phase, lasts from 2 h to a maximum of 12 h, and cows show signs of abdominal pain, grunting, bruxism, inappetence and tachycardia. Phase 2, the indolence phase, lasts until approximately day 3 of the disorder and the signs of pain disappear while the demeanour continues to be abnormal. Some cows may pick at their feed, they rarely ruminate and faeces in the rectum are replaced by mucus. Phase 3, the intoxication phase, lasts until approximately day 8 of the disorder and is characterised by marked deterioration of the health status. Signs of autointoxication and sepsis become evident, including increased body temperature and heart and respiratory rates, hyperaemic sclera, muddy mucous membranes, reduced skin surface temperature and sunken eyes. In the final stages of intussusception, cows become recumbent and die. In addition to the typical clinical signs of ileus, haemorrhagic to black faeces in the rectum, attributable to intestinal bleeding, is a diagnostic sign of small intestinal intussusception (SII). Descriptions of typical transrectal findings in cows include a firm mass at the pelvic inlet.^1^, ^21^, ^25^

Typical ultrasonographic findings are reduced or absent intestinal motility and dilated small intestines.^23^, ^24^ The intussusception has a bowel‐within‐bowel pattern, which appears in ultrasonograms as multiple parallel echoic lines that are separated by anechoic lines in longitudinal section and as elliptic to circular rings of varying echogenicity in cross‐section.^23^, ^24^, ^25^, ^26^

Surgery is the treatment of choice for intussusception.^1^ Several authors have described various surgical techniques.^1^, ^3^, ^5^, ^29^, ^30^, ^31^, ^32^ In cattle older than 6 months, a right‐flank approach in a standing position is ideal because it facilitates abdominal exploration compared with having the animal in a recumbent position.^1^ On the other hand, standing surgery involves the risk of cattle becoming recumbent during the procedure and subsequent abdominal contamination.^21^, ^33^ Manual reduction of the intussusception can be attempted when the small intestines appear sufficiently vital and changes are minimal.^1^ If the integrity of the intestines is questionable or cannot be assessed, intestinal resection and end‐to‐end anastomosis are recommended.^1^ Although SII is a well‐known disorder in cattle, the diagnostic findings, treatment and outcome have not been evaluated in a large number of cattle. Therefore, the objective of this study was to assess these features in 126 cattle from a referral population in Switzerland to determine the best tools for diagnosis and therapy in the early phases of the disorder.

## MATERIALS AND METHODS

The medical records of 126 cattle diagnosed with SII between January 1995 and December 2018 at the Veterinary College, University of Zurich, were analysed. The present work is based on two Master's theses.^34^, ^35^

### Inclusion and exclusion criteria

Only medical records of cattle that were at least 1 year of age and had SII, which involved the large colon in a few cases, were included provided that the diagnosis could be confirmed during laparotomy or postmortem examination.

### Cattle and history

There were 75.4% cows, 21.4% heifers and 3.2% bulls between 1 and 13 years (mean ± SD = 4.9 ± 2.7 years) of age. Breeds included Swiss Braunvieh (81.8%), Holstein (7.9%), Swiss Fleckvieh (7.1%) and others (3.2%). Fifty of the 122 female cattle (41%) were pregnant (median 20.5 weeks). The last calving date of 45 cows was between 3 and 37 weeks. At the time of admission, the duration of illness ranged from 4 h to 7 days (median 24 h). All cattle had anorexia or reduced feed intake. Signs of abdominal pain had occurred in 64.3% of cattle before admission.

### Clinical examination

Cattle underwent examination as previously described.^36^ The demeanour was considered mildly abnormal when a mild decrease in alertness and/or mild signs of colic (defined below) were present. A moderate decrease in alertness and sometimes occasional grunting and/or bruxism and marked signs of colic were observed in cattle with a moderately abnormal demeanour. Cattle with severely abnormal demeanour showed marked apathy and were sometimes recumbent and unable to rise.

Signs of pain were divided into non‐specific, somatic (parietal) and visceral (colic, abdominal pain). Non‐specific signs of pain included muscle fasciculations, bruxism and spontaneous grunting. Signs of somatic (parietal) pain were a tense abdominal wall, arching of the back and a tucked‐up abdomen. Visceral (colic, abdominal pain) signs consisted of shifting of weight in the hind limbs, lordosis, restlessness, kicking at the abdomen, sweating, tail swishing, frequent lying down and rising.

The number and severity of signs of colic/abdominal pain were determined. Signs of mild colic included mild restlessness, shifting of weight in the hind limbs, looking at the flank, lifting the tail, lifting of individual limbs and tail swishing.^37^ Signs of moderate colic were moderate restlessness, brief periods of recumbency, kicking with the hind limbs, arching of the back and marked tail swishing. Signs of severe colic consisted of marked restlessness, frequent lying down and rising, sweating, grunting and violent kicking at the abdomen.^37^

Cattle were also assigned to colic, indolence and intoxication phases. The colic phase was the initial phase accompanied by the previously described signs of pain. The indolence phase followed the colic phase and was characterised by apathy and a markedly abnormal demeanour. The last phase was intoxication, in which cattle had tachycardia, congested scleral blood vessels, markedly pale mucous membranes, cool skin surface temperature, sunken eyes and a dry muzzle.^37^

### Laboratory analyses

Blood and rumen fluid samples were analysed as previously described.^36^

### Ultrasonographic examination of the abdomen

The abdomen of 123 cattle was scanned from the right side as previously described.^24^

### Diagnosis

A tentative diagnosis of ileus was made in cattle that had a history of abdominal pain or in which such signs were evident on admission, combined with little or no faeces in the rectum. Ileus was definitively diagnosed when dilated small intestines and/or taut bands of mesentery were palpated transrectally; ileus attributable to intussusception was diagnosed when an additional firm mass could be palpated.

The ultrasonographic diagnosis of ileus was based on finding dilated loops of small intestines that had a diameter greater than 3.5 cm with absent or greatly reduced intestinal motility. The ultrasonographic diagnosis of ileus attributable to intussusception was based on the sonographic visualisation of the typical bowel‐within‐bowel pattern.^24^, ^25^ A definitive diagnosis was confirmed during laparotomy and/or postmortem examination.

### Laparotomy

A right‐flank laparotomy was carried out after a proximal paravertebral block of the last thoracic and the first two lumbar spinal nerves. Exploratory laparotomy was performed via an approximately 25–30 cm vertical incision placed in the mid‐paralumbar fossa. After routine exploration, the supraomental recess was carefully examined for dilated bowel parts, fibrin and signs of adhesion formation. A protective drape was placed in the laparotomy wound and then the affected bowel part was exteriorised if the location of the intussusception allowed it.

When resection anastomosis was deemed possible, the resection sites were determined and local anaesthetic was injected close to the vessels of the respective mesentery. Vessels were then ligated with braided absorbable suture material and two bowel clamps were placed at the level of each resection site. The bowel was transected between the bowel clamps using a scalpel and the mesentery was transected with scissors. An end‐to‐end anastomosis was performed using size 2.0 monofilament suture material in a simple continuous pattern, which was interrupted at four sites to avoid a purse string effect. A second suture was usually placed using the same material in a Cushing or Lembert pattern. After anastomosis, the mesenteric defect was closed with a simple continuous suture pattern using an absorbable polyfilament material. After liberal lavage of the anastomosis site, the bowel was repositioned into the abdomen. Using sterile instruments, new gowns and fresh gloves, the abdomen was closed in a routine fashion.

### Postoperative treatment

Cattle were fasted for at least 24 h postoperatively before feeding was gradually resumed. Medical treatment included antibiotics, fluid therapy, pain control and electrolyte replacement. Antibiotic treatment included penicillin G procaine (12,000 IU/kg bodyweight [BW]) or amoxicillin (7 mg/kg BW) given intramuscularly for 1–9 days (median 4 days). Seventy‐six cattle received a daily injection of flunixin meglumine (1 mg/kg BW) or metamizole (35 mg/kg BW) for 1–6 days (median 3 days). All cattle received 10 L of a solution containing 50 g glucose and 9 g sodium chloride per litre daily for 1–12 days (median 3 days) administered as a slow intravenous drip via an indwelling jugular vein catheter. Forty‐eight cows with hypocalcaemia (calcium less than 2.0 mmol/L) received 500 mL of 40% calcium borogluconate intravenously for 1–6 days (mean 1 day). Hypokalaemia (potassium less than 4.0 mmol/L) was treated in 53 cattle with daily oral doses of 60–100 g of potassium chloride until normokalaemia occurred (1–6 days, median 2 days). Cows with hypophosphataemia (inorganic phosphorus less than 1.0 mmol/L) or hypomagnesaemia (magnesium less than 0.7 mmol/L) were treated orally with sodium dihydrogen phosphate and/or magnesium oxide.

Prokinetic drugs were administered to 54 cattle for a duration of 1–9 days (median 4 days). Twenty‐eight cows received intramuscular metoclopramide and 26 received 40–45 mg neostigmine administered via continuous drip infusion. Seven cows received additional antiparasitic treatment.

### Euthanasia/slaughter

When indicated, cattle were slaughtered or they were euthanased using pentobarbital (80 mg/kg BW).

### Postmortem examination

All cattle that died or were euthanased underwent postmortem examination.

### Statistical analyses

The program SPSS Statistics 25.0 (IBM, Armonk, NY, USA) was used for analysis. Frequencies were determined for all variables and the Shapiro–Wilk test was used to test the data for normality. Means ± SDs were calculated for normally distributed data; medians (with 25th–75th percentiles) were calculated for non‐normally distributed data. The variables heart rate and rectal temperature over time (days 0–7) were analysed using the general linear model, choosing ANOVA with repeated measures and replacing polynomial contrasts with difference. Differences between the frequency distributions of the colic, indolence and intoxication phases were analysed using the chi‐square and the Bonferroni post hoc tests. Differences between the medians and means were analysed using the Kruskal–Wallis test (medians) and ANOVA (means). A value of *p* less than 0.05 was considered significant.

## RESULTS

### Demeanour, abdominal contour and signs of pain

The demeanour was mildly to severely abnormal in 97.6% of the cattle; 4% were recumbent and 16.7% had abdominal distension (Table 1).

**TABLE 1:** Demeanour, contour of the abdomen and signs of pain in cattle with small intestine intussusception (frequency distribution).

Variable	Finding	Number of cattle	Percent
Demeanour	Normal	3	2.4
	Mildly abnormal	13	10.3
	Moderately abnormal	84	66.7
	Severely abnormal	26	20.6
Abdomen	Bilateral abdominal distension	19	15.1
	Unilateral abdominal distension	1	0.8
	‘Papple’‐shaped abdominal contour	1	0.8
Non‐specific signs of pain	None	93	73.8
	Muscle fasciculations	13	10.3
	Bruxism	12	9.5
	Grunting	4	3.2
	Combinations of these signs	4	3.2
Signs of parietal pain	None	55	43.6
	Abdominal guarding	67	53.2
	Arching of the back	1	0.8
	Combinations of these signs	3	2.4
Stage	Colic stage	59	46.8
	Indolence stage	59	46.8
	Intoxication stage	8	6.4
Signs of visceral pain^a^	None	67	53.2
	Lordosis	33	26.2
	Treading	23	18.3
	Restlessness	10	7.9
	Kicking at the abdomen	7	5.6
	Tail swishing	2	1.6
	Frequent lying down and rising	2	1.6
Number of signs of visceral pain	No signs	67	53.2
	One sign	32	25.4
	Two signs	13	10.3
	Three signs	4	3.2
	Four signs	2	1.6
	Not recorded	8	6.3
Degree of visceral pain	No signs of visceral pain	67	53.2
	Mild	45	35.7
	Moderate	13	10.3
	Severe	1	0.8

^a^The total number of cattle was 144 because 18 had more than one sign of visceral pain.

Non‐specific signs of pain were recorded in 26.2% of the cattle and included muscle fasciculations, bruxism, grunting and combinations of these signs. Signs of parietal pain, including abdominal guarding, arching of the back and combinations of these signs were observed in 56.4% of cattle.

The colic phase was recorded in 46.8% of cattle, the indolent phase in 46.8% and the intoxication phase in 6.4%. Signs of visceral pain included lordosis, treading, restlessness, kicking at the abdomen, tail swishing, frequent lying down and rising. In 25.4% of cattle, there was one sign of visceral pain, 10.3% had two and 4.8% had three or four signs. Visceral pain was judged to be mild to severe.

### Heart and respiratory rates and rectal temperature

The heart and respiratory rates, as well as the rectal temperature, were not normally distributed. The most common abnormalities were decreased rectal temperature (61.6%), tachycardia (38.1%) and tachypnoea (28.0%) (Table 2).

**TABLE 2:** Clinical findings in cattle with small intestine intussusception (median, 25th–75th percentiles, frequency distribution)

Variable	Finding	Number of cattle	Percent
Rectal temperature (*n* = 125, 38.3°C, 37.8°C–38.6°C)	Normal (38.5–39.0)	38	30.4
	Decreased (36.0–38.4)	77	61.6
	Mildly increased (39.1–39.5)	9	7.2
	Moderately increased (39.6–40.0)	1	0.8
Heart rate (*n* = 126, 80 bpm, 68–91)	Normal (60–80)	67	53.2
	Decreased (44–59)	11	8.7
	Mildly increased (81–100)	37	29.4
	Moderately increased (101–120)	10	7.9
	Severely increased (121–148)	1	0.8
Respiratory rate (*n* = 125, 20 breaths/min, 20–28)	Normal (15–25)	89	71.2
	Decreased (12)	1	0.8
	Increased (26–80)	35	28.0
Rumen motility (*n* = 126)	Normal	3	2.4
	Decreased	73	57.9
	Absent	50	39.7
Foreign body tests (*n* = 120)	All negative	94	78.4
	At least one test positive^a^	26	21.4
BSA and PSA on the left side (*n* = 126)	Both negative (normal)	122	96.8
	Only BSA positive	1	0.8
	Only PSA positive	1	0.8
	Both tests positive	2	1.6
BSA and PSA on the right side (*n* = 126)	Both negative (normal)	43	34.1
	Only BSA positive	56	44.4
	Only PSA positive	5	4.0
	Both tests positive	22	17.5
Intestinal motility (*n* = 126)	Normal	8	6.3
	Decreased	84	66.7
	Absent	34	27.0
Rectal findings^b^ (*n* = 126)	Normal findings	23	18.3
	Rumen dilated	47	37.3
	Dilated loops of small intestines	31	24.6
	Empty loops of small intestines	23	18.3
	Intussusception palpable	2	1.6
	Miscellaneous abnormal findings	36	28.6
Faeces, amount (*n* = 126)	Normal	5	4.0
	Faecal output reduced	77	61.1
	Empty rectum	44	34.9
Faeces, degree of comminution (*n* = 122)	Normal (well digested)	66	54.1
	Moderately digested	7	5.7
	Poorly digested	5	4.1
	Rectum empty	44	36.1
Faeces, consistency (*n* = 124)	Normal	29	23.4
	Thick pulpy	23	18.5
	Greasy or pasty	21	16.9
	Thin pulpy	6	4.8
	Liquid	1	0.8
	Rectum empty	44	35.6
Faeces, colour and abnormal contents^c^ (*n* = 126)	Dark to black	38	30.2
	Blood	58	46.0
	Mucus	41	32.5
	Fibrin	7	5.6

Abbreviations: BSA, ballottement and simultaneous auscultation; PSA, percussion and simultaneous auscultation.

^a^ Positive: at least three of four attempts elicited a grunt.

^b^ The total number of findings was 162 (128.7%) because 42 cattle had more than one abnormal transrectal rectal finding.

^c^ The total number of findings was 144 (114.3 %) because 24 cattle had more than one abnormal faecal finding.

### Digestive tract

Rumen motility was reduced or absent in 97.6% of the cattle (Table 2). Ballottement and simultaneous auscultation (BSA) and percussion and simultaneous auscultation (PSA) were positive on the right side in 65.9% of the cattle. Intestinal motility was reduced or absent in 93.7% of the cattle. The most common abnormal findings of transrectal palpation were a dilated rumen and dilated small intestines. The intussusception could be palpated in two cows. Other findings (*n* = 5) were caecal dilatation, dilatation of the spiral colon, taut bands of mesentery and juxtaposition of empty and dilated loops of small intestines. In most cattle (96.0%), the rectum contained only small amounts of faeces or was empty. Faecal colour was dark to black in 30.2% of the cattle and consistency varied from liquid to pulpy to thick pulpy. Abnormal faecal contents included blood, mucus and fibrin.

### Other clinical findings

Other clinical abnormalities were reduced skin elasticity (76.2%), sunken eyes (68.8%), hyperaemic scleral vessels (60.3%), reduced skin surface temperature (60.2%), dry cool muzzle (57.9%), prolonged capillary refill time (40.8%), ammonia‐like breath (25.4%), pale oral mucosa (23.2%), cold ears (11.4%) and droopy ears (6.3%).

### Urinalysis

Urine pH ranged from 5.0 to 9.0 and was acidic (6.0 or less) in 29.7% and alkaline (greater than 8.0) in 22.5% of the cattle. The specific gravity ranged from 1.007 to 1.065; in 12.4%, it was less than 1.020 and in 17.1%, it was greater than 1.040. Glucosuria occurred in 49.5% of 111 cattle, haemoglobinuria/haematuria in 48.6%, proteinuria in 5.4% and ketonuria in 3.6%.

### Laboratory findings

Only the haematocrit, total protein concentration and venous blood pH were normally distributed (Table 3). The principal abnormalities were hypokalaemia (89.6%), hypocalcaemia (76.5%), positive base excess (72.9%), hypochloraemia (71.8%), azotaemia (62.1%) and haemoconcentration (61.1%). Rumen chloride was increased in 37.9% of the cattle.

**TABLE 3:** Laboratory findings in cattle with intussusception

Variable (median, 25th–75th percentiles) unless otherwise specified^a^	Finding	Number of cows	Percent
Haematocrit (*n* = 126) (37.5 ± 5.6%)^a^	Normal (30%–35%)	41	32.6
	Decreased (23%–29%)	8	6.3
	Increased (36%–58%)	77	61.1
Total leukocyte count (*n* = 124) (10,000/μL, 7425–13,000)	Normal (5000–10,000)	54	43.5
	Decreased (700–4999)	10	8.1
	Increased (10,001–23,300)	60	48.4
Total protein (*n* = 126) (80.8 ± 10.6 g/L)^a^	Normal (60–80)	58	46.0
	Decreased (42–59)	3	2.4
	Increased (81–113)	65	51.6
Fibrinogen (*n* = 122) (6.0 g/L, 4.8–8.0)	Normal (4–7)	62	50.8
	Decreased (1.0–3.9)	11	9.0
	Increased (7.1–16.0)	49	40.2
Urea (*n* = 124) (7.8 mmol/L, 5.6–11.9)	Normal (2.4–6.5)	46	37.1
	Decreased (0.7–2.3)	1	0.8
	Increased (6.6–35.8)	77	62.1
Bilirubin (μmol/L) (*n* = 125) (4.4 μmol/L, 2.5–6.9)	Normal (1.5–6.5)	84	67.2
	Decreased (0.1–1.4)	8	6.4
	Increased (6.6–27.4)	33	26.4
Aspartate aminotransferase (*n* = 125) (85 U/L, 72–116)	Normal (10–103)	85	68.0
	Increased (104–1104)	40	32.0
γ‐Glutamyl transferase (*n* = 125) (22 U/L, 17–29)	Normal (9–30)	105	84.0
	Increased (31–131)	20	16.0
Glutamate dehydrogenase (*n* = 65) (10.5 U/L, 7.8–16.2)	Normal (4–18)	53	81.5
	Increased (19–81)	12	18.5
Calcium (*n* = 51) (2.00 mmol/L, 2.00–2.29)	Normal (2.30–2.60)	9	17.6
	Decreased (1.00–2.29)	39	76.5
	Increased (2.61–3.26)	3	5.9
Magnesium (*n* = 51) (1.00 mmol/L, 1.00–1.56)	Normal (0.80–1.00)	29	56.9
	Increased (1.01–2.00)	22	43.1
Inorganic phosphate (*n* = 51) (2.00 mmol/L, 1.40–2.91)	Normal (1.30–2.40)	21	41.2
	Decreased (0.92–1.29)	12	23.5
	Increased (2.41–4.00)	18	35.3
Chloride (*n* = 124) (89.5 mmol/L, 82.3–97.0)	Normal (96–105)	34	27.4
	Decreased (61–95)	89	71.8
	Increased (106–108)	1	0.8
Potassium (*n* = 125) (3.20 mmol/L, 2.70–3.55)	Normal (4.0–5.0)	10	8.0
	Decreased (2.0–3.9)	112	89.6
	Increased (5.2–6.5)	3	2.4
Blood pH (*n* = 107) (7.44 ± 0.07)^a^	Normal (7.41–7.45)	25	23.4
	Decreased (7.25–7.40)	40	37.4
	Increased (7.46–7.59)	42	39.2
pCO_2_ (*n* = 107) (46.8 mmHg, 42.4–51.9)	Normal (35.0–45.0)	39	36.4
	Decreased (34.0–34.9)	2	1.9
	Increased (45.1–71.2)	66	61.7
Bicarbonate (*n* = 107) (29.9 mmol/L, 25.4–35.1)	Normal (20.0–30.0)	52	48.6
	Decreased (18.4–19.9)	2	1.9
	Increased (30.1–62.3)	53	49.5
Base excess (*n* = 107) (7.7 mmol/L, 1.4–11.3)	Normal (–2 to +2)	21	19.6
	Decreased (–7.1 to –2.1)	8	7.5
	Increased (+2.1 to +35)	78	72.9
Rumen chloride (*n* = 103) (24 mmol/L, 19–29)	Normal (≤25)	64	62.1
	Increased (26–55)	39	37.9

^a^Mean ± standard deviation for normally distributed variables.

### Clinical and laboratory findings in relation to the stage of disease

Severe congestion of the scleral blood vessels occurred less often (*p* < 0.01) in the colic phase (8.5%, *n* = 5) than in the indolence phase (13.6%, *n* = 8) and intoxication phase (62.5%, *n* = 5). Spontaneous grunting also occurred less frequently in the colic phase (1.7%) than in the indolence (3.4%) and intoxication (25.0%) phases. The median rectal temperature was 38.4°C in the colic phase, 38.3°C in the indolence phase and 37.5°C in the intoxication phase (*p* < 0.05) (Figure S1A). The median values for serum urea (Figure S1B) and pCO_2_ (Figure S1C) were lowest in the colic phase and highest in the indolence phase (*p* < 0.05). The concentration of serum chloride (Figure S1D) and urine pH were highest in the colic phase (8.0) and lowest in the intoxication phase (5.0).

### Ultrasonographic findings

The principal findings were reduced or absent small intestinal motility (98.2%), dilated loops of small intestines (96.0%) and fluid between intestinal loops (65.9%) (Table 4). In 8.1% of the cattle, dilated intestinal loops were seen in juxtaposition with empty loops. The intussusception was visualised in 5.7% of the cattle and appeared as the typical bowel‐within‐bowel pattern (Figure S2).^24^ The abomasum was dilated in 28.5% of the cattle because of retrograde accumulation of ingesta.

**TABLE 4:** Ultrasonographic findings in cattle with small intestine intussusception

Variable	Finding	Number of cattle	Percent
Intestinal motility (*n* = 113)	Normal	2	1.8
	Decreased	19	16.8
	Absent	92	81.4
Cross‐section of small intestines (*n* = 123)	Normal	5	4.0
	Dilated	118	96.0
Largest diameter of small intestines (cm) (*n* = 123)	Normal (2.7–3.5)	5	4.1
	Slightly dilated (3.6–4.0)	12	9.8
	Moderately dilated (4.1–6.0)	63	51.2
	Severely dilated (6.1–11.0)	32	26.0
	Diameter not determined	11	8.9
Empty poststenotic small intestines (*n* = 123)	Not visible	113	91.9
	Visible	10	8.1
Intestinal content (*n* = 123)	Not assessed	81	65.9
	Echoic	22	17.9
	Hypoechoic	6	4.9
	Echoic and hypoechoic	3	2.4
	Homogenous	7	5.7
	Heterogeneous	3	2.4
	Blood clots	1	0.8
Intestinal wall (*n* = 123)	Normal	118	95.9
	Thickened	5	4.1
Visualisation of intussusception (*n* = 123)	Not visible	116	94.3
	Visible	7	5.7
Fluid between intestinal loops (*n* = 123)	No fluid	42	34.1
	Fluid without fibrin	73	59.4
	Fluid with fibrin	8	6.5
Fibrin between intestinal loops (*n* = 123)	No fibrin between intestinal loops	118	95.9
	Fibrin between intestinal loops	5	4.1
Abomasal dilatation (*n* = 123)	Not dilated	88	71.5
	Dilated	35	28.5

### Intercurrent diseases

A total of 29.4% of cattle had one (19.9%), two (6.3%) or three (3.2%) diseases. The most frequent were gastrointestinal parasitism, mastitis, claw lesions, enteritis and fascioliasis.

### Diagnoses

Based on the clinical findings alone, a tentative diagnosis of ileus was made in 70.6% of the cattle, a definitive diagnosis of ileus in 25.4% and a diagnosis of ileus attributable to intussusception in 0.8% (Table 5). With the inclusion of ultrasonographic findings, which were available for 123 cattle, the rate of diagnosis of ileus increased significantly from 25.4% to 90.2% of the cattle. Furthermore, a diagnosis of ileus attributable to intussusception could be made in another 5.7%. Combining clinical and ultrasonographic findings allowed an increase in the rate of diagnosis of ileus to 95.2% (*n* = 120) and provided a correct diagnosis of intussusception in 12 of these cattle.

**TABLE 5:** Diagnoses in 126 cows with small intestine intussusception

Parameter	Clinical diagnosis (*n* = 126)	Ultrasonographic diagnosis (*n* = 123)	Diagnosis based on clinical and sonographic findings (*n* = 123)
Tentative diagnosis of ileus	70.6% (*n* = 89)	0	0
Definitive diagnosis of ileus	25.4% (*n* = 32)^a^	90.2% (*n* = 111)^1^ ^,b^	87.8% (*n* = 108)^c^
Diagnosis of ileus attributable to intussusception	0.8% (*n* = 1)	5.7% (*n* = 7)	9.8% (*n* = 12)
Other or no diagnosis	(3.2%) (*n* = 4)^2^	4.1% (*n* = 5)	2.4% (*n* = 3)

*Note*: Differences a:b and a:c (*p* < 0.01).

^1^ Eight also had peritonitis.

^2^ Dilatation of the caecum in one cow; no diagnosis in three cows.

### Treatment decisions and outcome

**FIGURE 1:**
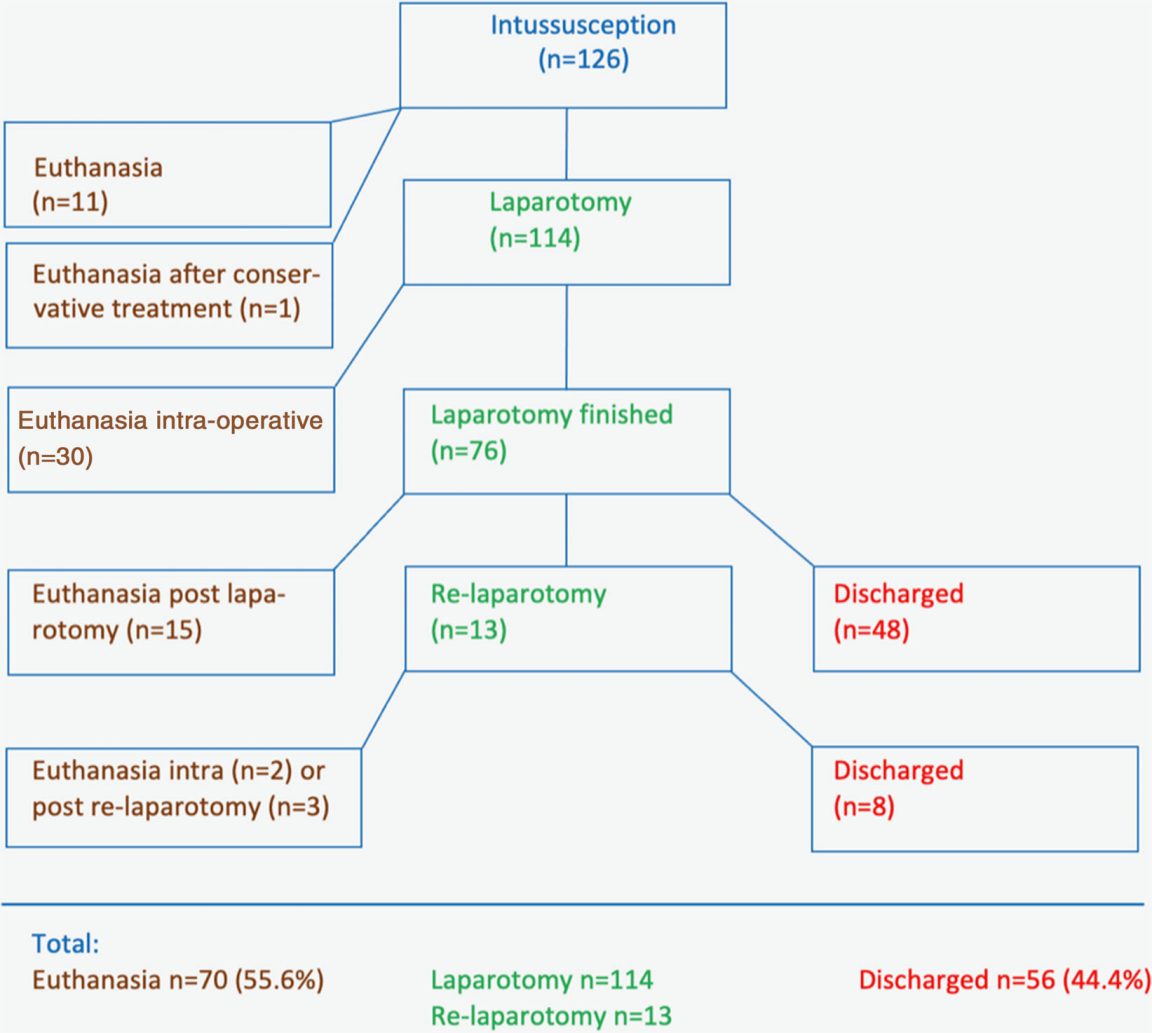
Treatment flowchart for 126 cattle with intussusception of the small intestines.

Eleven (8.7%) cows were euthanased or slaughtered after the diagnosis had been made because of a poor prognosis (Figure 1). Another cow with an initial diagnosis of fibrinous traumatic reticuloperitonitis was also euthanased after conservative treatment for 2 days (fibrinous reticuloperitonitis was confirmed during postmortem examination during which an additional intussusception was diagnosed).

Of 114 cattle that underwent laparotomy, 38 were euthanased intraoperatively; the operation was completed successfully in the remaining 76 cattle. In two of the latter, the intussusception was reduced manually; the remaining 74 underwent bowel resection and anastomosis; all received medical postoperative treatment for 3–12 days (median 3–4 days). Fifteen cattle were euthanased or died postoperatively, 13 were operated on a second time and 56 were discharged.

Thirteen cows did not eat or defaecate after surgery and therefore underwent relaparotomy; of these, five were euthanased during or after surgery and the remaining eight were discharged. Seven of the latter had intestinal atony and were treated by massaging the intestinal content from the non‐motile segments aborally; one underwent intestinal resection and anastomosis because of a recurring intussusception.

In summary, 114 (90.5%) cattle were treated surgically and 13 of these were operated on twice. Seventy (55.6%) cattle were euthanased or slaughtered and 56 (44.4%) were treated successfully and discharged. Fifty percent of cattle in the colic and indolence phases survived and no cattle survived from the intoxication phase.

### Location of the intussusception

The intussusception was jejunojejunal in 94.4% of the cattle and jejunoileal, jejunoileocaecal, ileocaecal or ileoileal in another 5.6% (Figure S3). In the cattle with jejunojejunal intussusception, the lesion was in the proximal, middle or distal section.

### Intraoperative complications and other surgical findings

Intraoperative complications occurred in 39.5% of the 114 operated cattle and included cows becoming recumbent with subsequent contamination of the intestines, contamination of the abdominal cavity during resection, haemorrhage, rupture of the bowel during surgical manipulation and suboptimal anastomosis. Other findings were mesenteric lesions (*n* = 23). Twenty cows had peritoneal effusion and another 20 had fibrinous adhesions. In six cattle, the mesentery or even the greater omentum was involved in the intussusception. Five cattle had a ruptured bowel and two cows had two or three intussusceptions.

### Outcome for the 56 successfully treated cattle (during hospitalisation)

**FIGURE 2:**
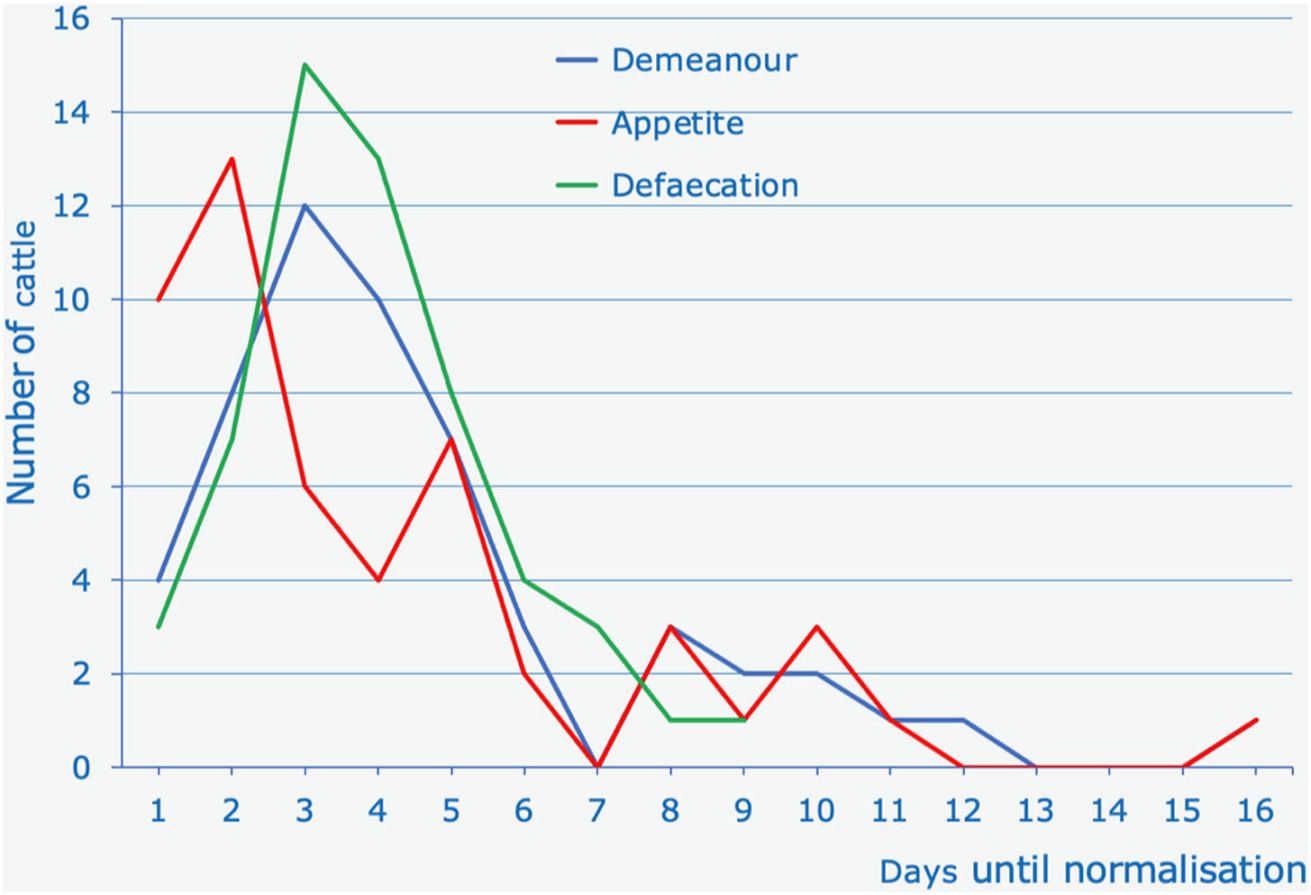
Normalisation of demeanour, appetite and defaecation in 56 cows after surgical treatment of small intestinal intussusception.

The demeanour and appetite of the successfully treated cattle returned to normal within 1–16 days (Figure 2) and normalisation of defaecation took 1–9 days. In a few cows, the demeanour (*n* = 2), appetite (*n* = 5) and defaecation (*n* = 1) did not return to normal during hospitalisation.

The rectal temperature increased within 7 days from 38.3°C to 38.7°C (medians, *p* < 0.01, Figure S4). It failed to return to normal during hospitalisation in seven cows (increased in three and decreased in four).

The median heart rate decreased within 7 days from 80 to 72 bpm (*p* > 0.05). It failed to return to normal during hospitalisation in two cows (increased in one and decreased in one other).

General health was good at discharge in 49 cows and suboptimal in the remaining seven cows.

### Long‐term outcome for the 56 successfully treated cattle

The long‐term outcome was assessed via a telephone follow‐up for 39 cattle 2 years after discharge and it was considered to be good in 31 cattle. One animal was slaughtered because of poor performance and another because of recurrence of intussusception. Six cows were slaughtered for economic reasons.

### Postmortem findings

The principal findings in 70 cattle that underwent postmortem examination were intussusception with haemorrhagic infarction, prestenotic intestinal dilatation, dilated abomasum and forestomachs and empty poststenotic intestines. In addition, five cows had a ruptured bowel, four had small intestinal volvulus, three had dehiscence of the anastomosis suture and peritonitis, two had an intestinal ulcer (one was perforated), one animal had necrosis of the anastomosis and one other had bowel obstruction at the site of anastomosis. Fourteen cattle had unclotted or coagulated blood in the intestines and seven had enteritis. Five cattle had localised peritonitis, five had generalised peritonitis and three had fascioliasis.

## DISCUSSION

A protracted course is typical for intestinal intussusception, presumably because of progressive occlusion of the intestinal lumen and impaired flow of intestinal contents.^1^, ^21^ The findings of the present study reflected the progressive course of this condition; approximately one‐third of all cattle (35.2%) had been ill for a minimum of 48 h and signs of abdominal pain were seen in less than half of all cattle (46.8%). The absence of colic in 53.2% of our cases is because the colic phase in cattle with ileus is very short at 12 h,^37^ and these cattle were already in the indolence and intoxication phases. Interestingly, signs of abdominal pain were usually mild and therefore easily missed; the most common signs were lordosis and treading, whereas more obvious signs such as kicking at the belly were rare. This was in agreement with observations by others who did not mention signs of colic^21^ or only mild signs.^6^, ^38^ The severity of the illness may be underestimated when no or only mild signs of abdominal pain are seen in cattle with intussusception. This in turn may result in cattle not being presented for veterinary examination until severe changes have occurred, leading to a poor prognosis.

Although many different disorders result in a positive BSA and/or PSA on the right side, this finding usually points to a serious gastrointestinal condition. Further diagnostic measures must be instituted if the aetiology cannot be determined. The results of BSA and/or PSA were positive on the right side in 65.9% of cattle with intussusception, which was much lower than test results in cows with abomasal volvulus (99.4%),^39^ right displaced abomasum (98.6%)^39^ and caecal dilatation/torsion (90%).^40^ We assume that cows with the latter conditions accumulate larger amounts of fluid and gas in the intestinal tract; therefore, positive BSA and PSA are more prevalent.

Transrectal palpation of dilated loops of small intestines supported a diagnosis of ileus in 24.6% of all cattle, while palpation of the actual intussusception was only possible in two cows. In contrast, a study of 48 cows with SII reported that dilated small intestines could be palpated transrectally in 50% of the cows and a firm mass that was thought to be the actual intussusception could be palpated in 22.9%.^21^ In another study, a mass suggestive of intussusception could be palpated transrectally in 13 (65%) of 20 cows.^38^ Transrectal palpation of an intussusception is rarely possible in cows in advanced pregnancy, very large cows or cows with an intussusception of the proximal jejunum. In addition, dilatation of the small intestines to the extent that they can be palpated transrectally may take 48 h or longer.^3^ In the present study, 105 (84%) cattle had been clinically ill for no longer than 48 h on admission, which may explain the relatively small number of cattle with dilated loops of small intestines on transrectal examination compared with other studies.^21^, ^38^ In the present study, 34.9% of the cattle had an empty rectum compared with 48.0%^3^ and 100% in other studies.^38^ We believe that these differences also reflect a shorter duration of illness in the cattle of the present study. Taken together, these considerations emphasise that ileus cannot be ruled out based on the presence of faeces in the rectum.

Characteristic ultrasonographic findings in cows with ileus are reduced or absent intestinal motility and dilated small intestines,^23^, ^24^, ^25^, ^26^ which were seen in 98.2% and 96.0% of our cases, respectively. However, the typical bowel‐within‐bowel pattern was only seen in 5.7% of the cattle. Other authors reported direct visualisation of the intussusception in four of six^25^ and in three of five cases.^26^ In our experience, the cause of ileus in cattle can rarely be detected ultrasonographically. In adult cattle, the intussusception may be too far from the abdominal wall to be visualised. A diagnosis is also difficult in cows in advanced pregnancy.^25^ Ultrasonography is an important adjunctive diagnostic technique in cows with ileus and increased our diagnostic rate from 25.4% (based on clinical findings alone) to 97.6%.

Indications of intussusception include signs of protracted ileus, transrectal palpation of the intussusception (rare) and visualisation of the intussusception as a bowel‐within‐bowel structure via ultrasonography (rare). Based on a discharge rate of 44.4%, ileus attributable to intussusception had a guarded prognosis. The long‐term outcome was good in most successfully treated cows.

## AUTHOR CONTRIBUTIONS

Ueli Braun initiated, planned and supervised the study and prepared the manuscript. Christian Gerspach and Karl Nuss were involved in revising the manuscript. Surgical treatment was performed by Karl Nuss and his assistants. Monika Hilbe was responsible for the postmortem examinations. Claudia Volz and Muriel Boesiger analysed the medical histories of the cows as part of their master's theses. All authors read and approved the final manuscript.

## CONFLICTS OF INTEREST STATEMENT

The authors declare they have no conflicts of interest.

## FUNDING INFORMATION

Not applicable since it was a retrospective analysis of medical records.

## ETHICS STATEMENT

Informed consent was gained from the owners of all cows before clinical, laboratory and ultrasonographic examinations were started.

## Supporting information



FIGURE S1 Rectal temperature (A), serum urea (B), pCO_2_ (C) and serum chloride (D) in the colic (*n* = 59), indolence (*n* = 59) and intoxication phases (*n* = 8).
FIGURE S2 Ultrasonographic (A) and postmortem findings (B) in a 7‐year‐old Brown Swiss cow with jejunal intussusception.
FIGURE S3 The location of the intussusception in 126 cattle.
FIGURE S4 Rectal temperature of 56 cows after surgical treatment of small intestinal intussusception.

## Data Availability

All data relevant to the study are included in the article. The data sets used and analysed during the current study are available from the corresponding author upon reasonable request.
