# Supplementary material for: A retrospective review of small intestinal intussusception in 126 cattle in Switzerland
Source: Vet Rec Open. 2023 Mar 28;10(1):e58. doi: 10.1002/vro2.58 (PMC10049975; doi:10.1002/vro2.58)
Supplement: Supplementary file 1 — FIGURE S1 Rectal temperature (A), serum urea (B), pCO2 (C) and serum chloride (D) in the colic (n = 59), indolence (n = 59) and intoxication phases (n = 8). FIGURE S2 Ultrasonographic (A) and postmortem findings (B) in a 7‐year‐old Brown Swiss cow with jejunal intussusception. FIGURE S3 The location of the intussusception in 126 cattle. FIGURE S4 Rectal temperature of 56 cows after surgical treatment of small intestinal intussusception. [file VRO2-10-e58-s001.pdf]

Supporting information

Figure S1 Rectal temperature (A), serum urea (B), pCO_2 (C) and serum chloride (D) in the colic (n=59), indolence (n=59) and intoxication phases (n=8)

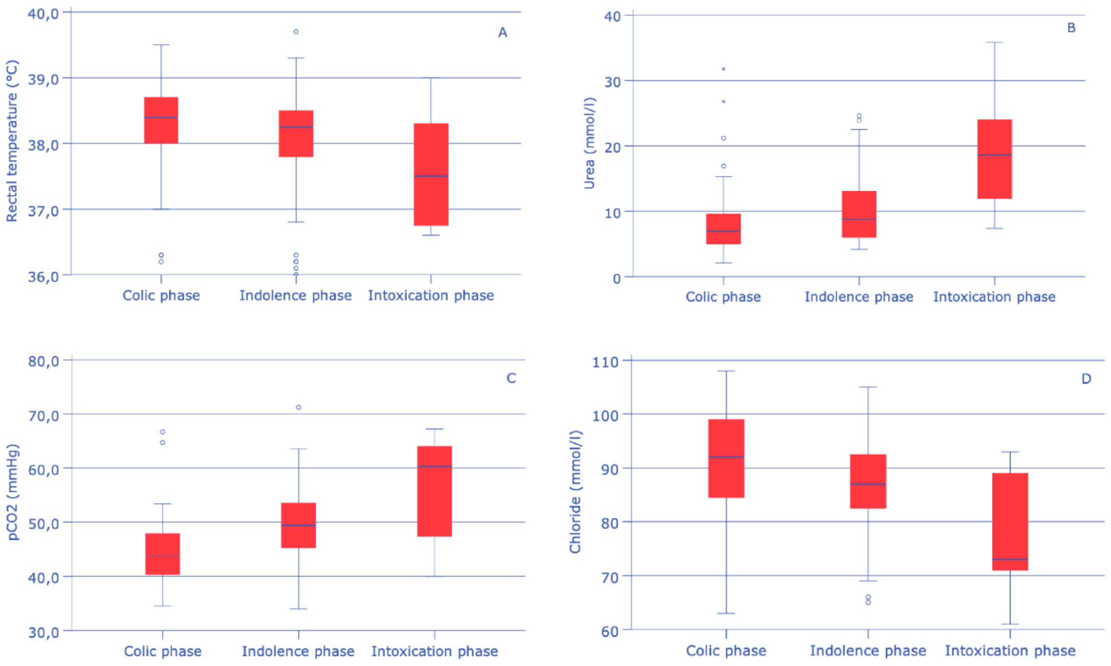

Within a box, the thick horizontal line shows the median. The top and bottom of the blue box represent the upper and lower quartiles, respectively. The distance between the top of the box and the top of the whisker shows the range of the top 25% of scores. Similarly, the distance between the bottom of the box and the end of the bottom whisker shows the range of the lowest 25% of scores. °=Outliers, * = Extreme outliers.

Figure S2 Ultrasonographic (A) and postmortem findings (B) in a 7-year-old Brown Swiss cow with jejunal intussusception

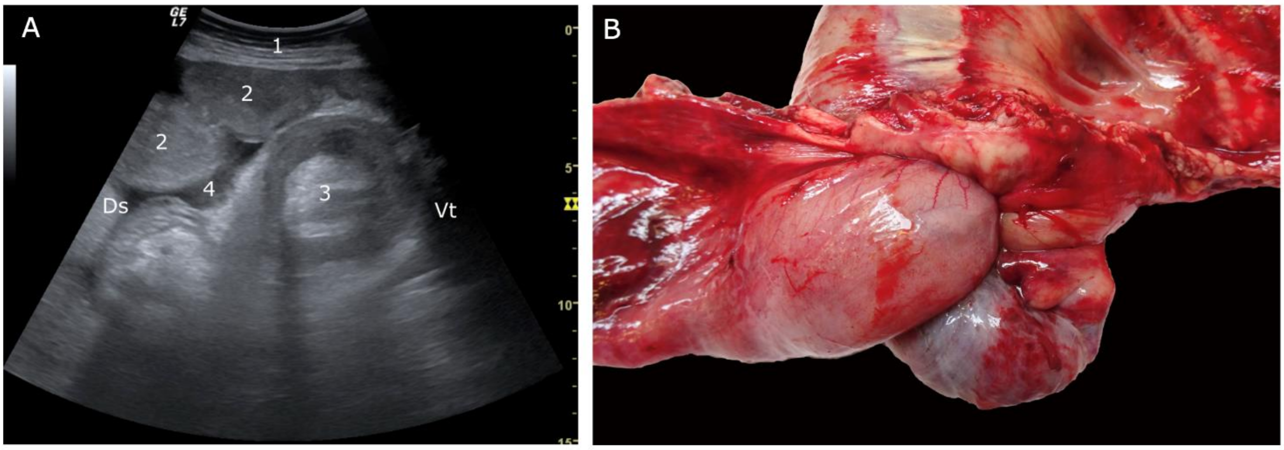

The ultrasonogram (A) shows a cross-section through the intussuscepted jejunum. The intussusception has the appearance of concentric rings. The postmortem preparation (B) shows the intussuscepted jejunum. 1 Abdominal wall, 2 Dilated loop of the jejunum in cross section. 3 Intussusception, 4 Fluid between the jejunal loops, Ds Dorsal, Vt Ventral.

Figure S3 Location of the intussusception in 126 cattle

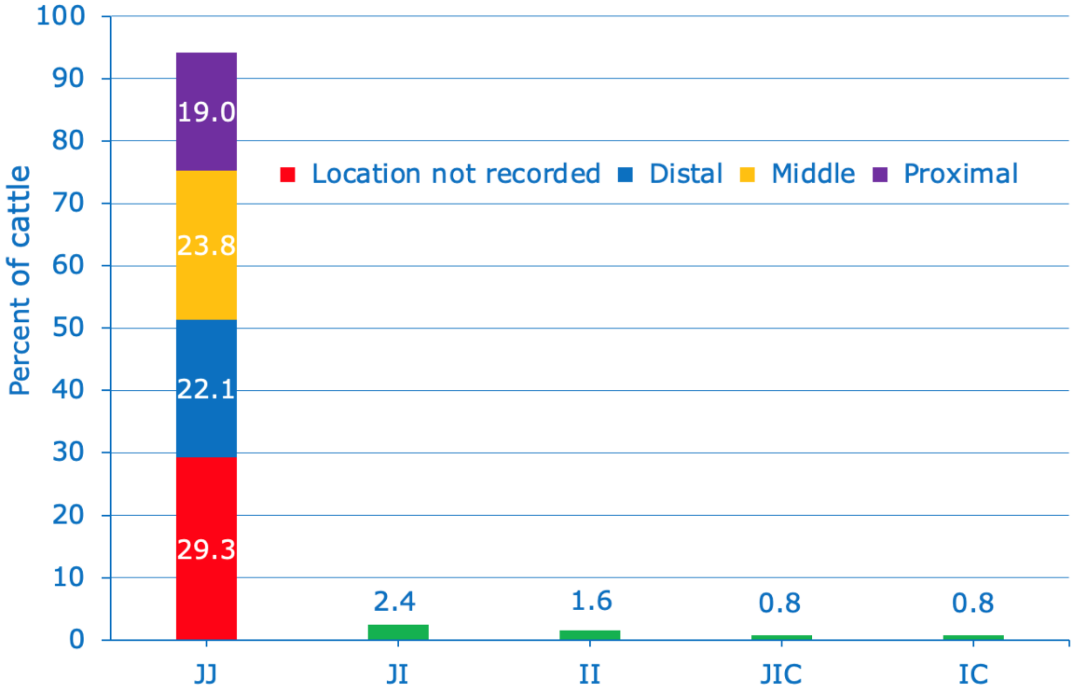

JJ Jejunojejunal, JI Jejunoileal, II Ileoileal, JIC Jejunoileocaecal, IC Ileocolic.

Figure S4 Rectal temperature of 56 cows after surgical treatment of small-intestinal intussusception (Day 0 n=126; Day 1 n=73, Day 2 n=69, Day 3 n=63, Day 4 n=60, Day 5 n=55, Day 6 n=45, Day 7 n=33

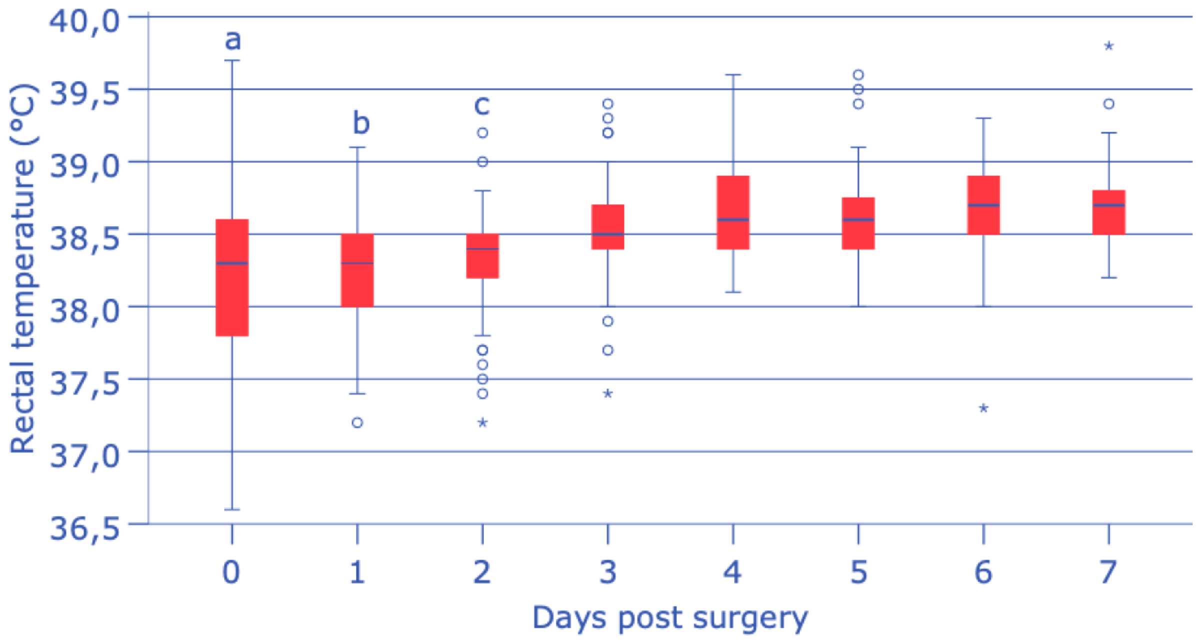

* = Extreme scores, ° = Outliers.
Because of the scale of the y-axis, four outliers (36.0, 36.1, 36.3, 36.3 °C) are not shown on Day 0 and one each is not shown on Days 3 (40.3 °C), 4 (40.2 °C) and 6 (40.1 °C).
[a] Different from Day 7 (P<0.05)
[b] Different from Days 3 to 7 (P<0.05)
[c] Different from Day 7 (P<0.05)
